# Advantages of a technique using two 50 degree arcs in simultaneous integrated boost radiotherapy for left-sidebreast cancer

**DOI:** 10.1038/s41598-017-15307-7

**Published:** 2017-11-07

**Authors:** Dan Xi, Yun Ding, Rui Hu, Wendong Gu, Jinming Mu, Qilin Li

**Affiliations:** 1 Department of Radiation Oncology, The Third Affiliated Hospital of Soochow University, The First Peoples’ Hospital of Changzhou, Changzhou, 213003 China; 2 Department of Radiation Oncology, Affiliated Suzhou Hospital of Nanjing Medical University, Suzhou Municipal Hospital, Suzhou, 213003 China

## Abstract

This study evaluated radiotherapy techniques with 15 cases for simultaneous integrated boost to treat whole left breast and tumor bed following breast conserving surgery. Treatment plans were generated using three techniques: volumetric modulated arc therapy (VMAT) with a partial arc of 190° (1ARC), VMAT with two tangential mini-arcs of 50° each (2TARC) and intensity modulated radiation therapy with four fixed angle fields (4IMRT). Dosimetric parameters for the whole breast (Target), the boost tumor bed (Boost), and surrounding normal organs were compared. Chair Index (CHI) was introduced to evaluate the dose homogeneity in Target given the two levels of prescription dose. The dose coverage in Target was better in 1ARC and 2TARC than that in 4IMRT. The mean CHI in 1ARC (2.47) and 2TARC (2.62) were higher than that in 4IMRT (1.71, p < 0.05), and this indicated the dose homogeneity of Target was better in 1ARC and 2TARC than that in 4IMRT. The mean doses to right lung, and contralateral breast in 4IMRT were lower than those in 2TARC but the differences between them were small. 2TARC was better than 4IMRT with respect to the dose to ipsilateral lung and heart. Overall, 2TARC was optimal among three techniques.

## Introduction

Adjuvant radiotherapy (RT) is an essential part after the breast conserving surgery (BCS) for breast cancer patients^1–5^. Studies have shown that RT after BCS provides the same survival benefits as treated by radical mastectomy for early-stage breast cancer. Good cosmetic result is the main advantage of breast conserving treatment. The standard regimen for radiotherapy is to irradiate the whole breast (WBI) 45–50 Gy in about 5weeks, followed by a boost treatment to the tumor bed for additional another 10–16 Gy. The entire radiation treatment may last 6–7 weeks. This regimen is also called sequential boost technique (SBT)^2,6,7^.

An alternative method to SBT, is the simultaneous integrated boost technique (SIB)^7–9^, in which 60–66 Gy is prescribed to the tumor bed and to be delivered simultaneously with the standard treatment to the whole breast. The overall treatment time by SIB is reduced. In addition to lowering the cost of treatment, SIB may be beneficial in term of increased tumor control probability (TCP) due to an increased fractional dose to the tumor bed. Modern radiotherapy techniques, such as intensity-modulated radiation therapy (IMRT) and volumetric modulated arc therapy (VMAT), can be used to implement SIB technique straight forwardly.

The use of VMAT for WBI SIB has been studied for several years^10–13^. However, few studies were focused on comparing different VMAT and IMRT techniques for WBI SIB^7,8^, but there has been numerous reports on VMAT applied to the WBI only^2,3,5,6,14,15^. These results are inconclusive. For example, both Zhao *et al*.^6^ and Badakhshi *et al*.^2^ found that two-field IMRT was more suitable than VMAT for WBI, but Yu *et al*.^15^ concluded that two-small-arc VMAT was a better irradiation technique for WBI when compared to four- field IMRT. Similarly for VMAT for WBI SIB, Wu *et al*.^7^ concluded that IMRT may be more suitable for the SIB than VMAT, but Aly *et al*.^8^ found that VMAT ranked higher than IMRT with respect to overall assessment of plan qualities. In order to investigate the performance of VMAT for WBI SIB, we decided to compare the dosimetric features among three techniques: one partial arc VMAT, two tangential small arcs VMAT and four-field IMRT. In addition, patients with the tumor in the inner quadrant of breast were investigated separately.

## Methods and Materials

In this study, the treatment plan quality was evaluated by a set of pre-defined dosimetric parameters for different RT techniques. This study was given IRB approval by the First People’s Hospital of Changzhou. Written informed consent was obtained from the patients before treatment. The methods used were in compliance with the guidelines in the Declaration of Helsinki. Fifteen early stage breast cancer patients with left-side lesion treated in our institution were retrospectively selected. Four of the studied patients had inner quadrant tumor, which were included in additional separate analyses. Patients were treated in supine position on a wing board with arms positioned above the head. Planning CT scans with 3 mm slice thickness were acquired using a Siemens Somatom Sensation Open 40-slice CT scanner (Siemens Medical Solutions, Forchheim, Germany). The image data sets were imported into a Monaco Treatment Planning System (Monaco version3.3, Elekta AB, Stockholm, Sweden) for planning. An X-ray voxel Monte Carlo algorithm^16^ was used for dose calculation with grid size: 3 × 3 × 3 mm^3^.

### Contour Delineation

The clinical target volume (CTV) and the tumor-bed were delineated by two experienced radiation oncologists. The CTV included whole glandular breast tissue cropped 5mm inside the skin contour. The plan tumor volume, labeled as PTVbreast, was created with a 5 mm margin on the CTV expansion and also cropped 5 mm to the skin contour. The organs at risk included spinal cord, right and left lung, right breast and heart. The tumor-bed was delineated with guidance of the surgical scar, surgical clips, and images of pre- and post-operation. The boost target volume, labeled as PTVboost, was generated by adding a 5 mm margin to the tumor-bed and cropped 5 mm to the skin contour. PTVboost was completely inside the PTVbreast.

### Treatment planning

**Figure 1:**
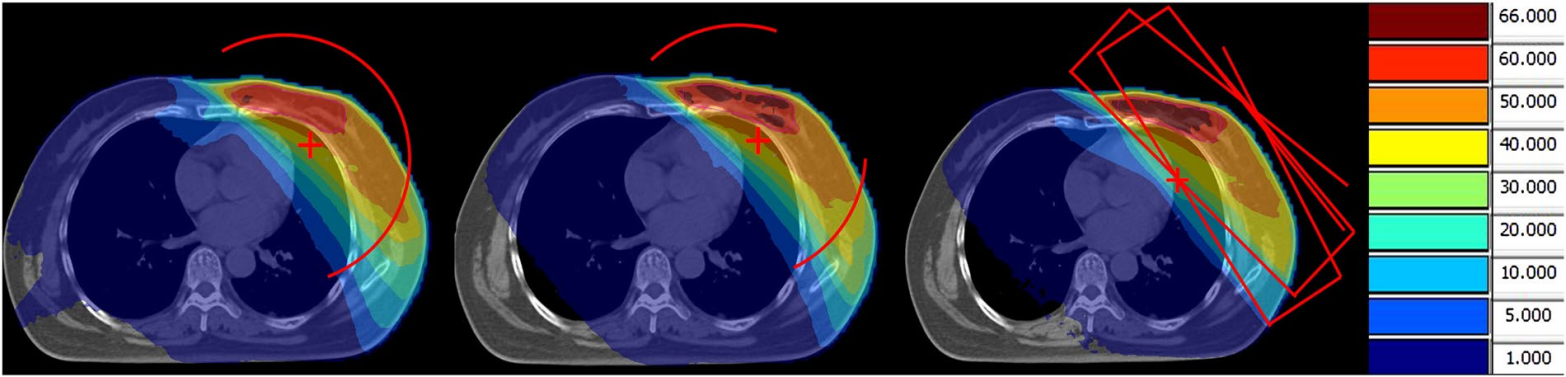
Dose distribution in a selected transversal plane and the beam arrangement in three techniques.

The treatment plans were re-designed with three different techniques: one partial arc VMAT (denoted as 1ARC), two tangential mini-arcs VMAT (denoted as 2TARC) and four-field IMRT (4IMRT). 1ARC used around 190° (185°–205°) partial arc. 2TARC used two tangential, around 50° (48°–56°) partial arcs (medial and lateral arcs; the range (min-max) was denoted in the brackets.). 4IMRT employed four coplanar, tangential beams with carefully selected gantry angles in order to achieve the best plan, and there was a limit of maximum 15 segments per field. Figure 1 show the arc arrangements and beam directions for the three techniques. The prescription for PTVbreast was 50 Gy and PTVboost 60 Gy in 25 fraction. The planning objective was deliver at least 95% of the prescribed dose to 95% of the target volumes (D95 ≥ 95%). The plans were generated using a 6MV photon with an Axesse^®^ linac (Elekta AB, Stockholm, Sweden).

### Plan Evaluation

For PTVbreast and PTVboost, heterogeneity index (HI) and conformity index (CI) were calculated.

CI is the product:

1 $$CI=\frac{{{\rm{V}}}_{{\rm{Tref}}}}{{{\rm{V}}}_{{\rm{T}}}}\times \frac{{{\rm{V}}}_{{\rm{Tref}}}}{{{\rm{V}}}_{{\rm{ref}}}}$$

where V_Tref_ is the target volume covered by prescription dose, V_T_ is target volume, and V_ref_ is the total volume covered by the prescription dose. It is the product of two volume ratios, one reflects the coverage of the target volume by the prescription dose and the other reflects the degree of conformation of the high dose volume to the target. The ideal CI value is 1 (CI ≤ 1).

HI is defined by:

2 $$HI=\frac{{{\rm{D}}}_{{\rm{2}}}-{D}_{98}}{{{\rm{D}}}_{{\rm{95}}}}\times 100 \% $$

where D_2_, D_95_, D_98_ are the irradiation dose to 2%, 95%, 98% of the target volume, respectively. Smaller HI value indicates a better dose homogeneity in the target volume (HI ≥ 0).

**Figure 2:**
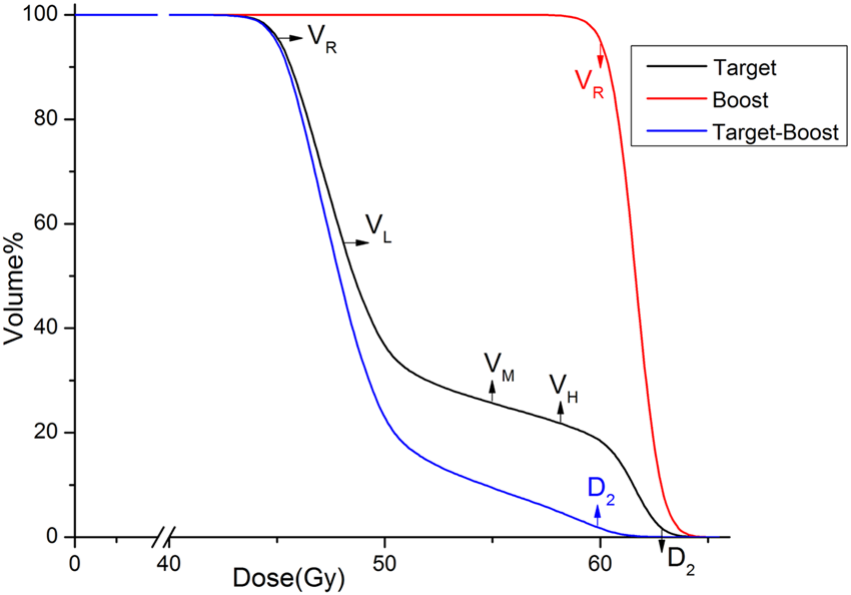
A typical dose-volume histogram of target and boost volumes. D_2_ represents the dose to 2% of the volume. V_R_, V_L_, V_M_, and V_H_ are the percentage volume of the target that receives dose of R, L, M and H respectively. R is the prescription dose to the volume.

For SIB technique the evaluation of the target homogeneity with HI becomes challenging, since D_2_ is much higher than D_98_ because of two levels of prescription dose (Fig. 2). For this reason a Chair Index (CHI) was introduced to characterize the dose homogeneity for the target volumes in SIB. In this case, the ideal cumulative dose volume histogram (DVH) curve for the target is not a vertical line but shapes like an outline of a chair.

CHI is defined by:

3 $$CHI=\frac{{V}_{R}-{V}_{L}}{{V}_{M}-{V}_{H}}$$

where Vx was the percent of the target volume received X dose. The subscripts R, L, M and H were the prescription dose of the target, reference low dose, medium dose and high dose values, and R < L < M < H < the prescription dose of the boost volume (Fig. 2). The reference doses L, M, and H were specified as the following:

1. M was the mean of the prescription dose of the main target and of the boost volume. M should be in the plateau of the target cumulative DVH curve.
2. L was around 7% higher than the presciption dose of the target volume based on the recommendation of ICRU Report 50^17^ that the dose coverage of the target be kept within +7% and −5% of the prescribed dose. V_L_ reflected the first fall-off of the Target cumulative DVH curve. The lower the V_L_, the steeper the first fall-off of the Target curve. The M and L values so selected ensure that V_L_ was greater than V_M_.
3. H was around 5% lower than the prescription dose to boost volume and also based on the recommendation of ICRU Report 50^17^. Obviously, V_H_ is less than V_M._

A higher CHI indicates a better dose distribution inside the target excluding the boost volume.

In this study, L, M and H were set as 53, 55 and 58 Gy respectively.

Additionally, the volume of the target volume that receives 107% of the prescription dose (V107%) was used to report the size of the hot spots.

For the organs at risk, the dosimetric parameters evaluated were the following: maximum dose (Dmax) of the Spinal cord and Breast_Right, V5, V10, V20, and mean dose (Dmean) for Lung_Left and Heart; V5 and Dmean for the Lung_Right, Dmean and Dmax of Breast_Right.

### Statistical analysis

All statistical tests were performed using the SPSS software (version 13.0, SPSS Inc., Chicago, IL, USA). Paired T Test or Wilcoxon rank sum test was used to evaluate plan quality differences with different planning techniques. P value less than 0.05 is considered statistically significant.

## Results

### 1ARC and 2TARC were superior to 4IMRT in the targets dosimetricparameters

Table 1 lists the dosimetric parameters used for plan quality evaluation. The objectives for target dose coverage with prescriptions were all achieved. For the boost volume, PTVboost, the dose coverage in terms of HI, CI and D_2_ were statistically better for 1ARC or 2TARC than 4IMRT. The average HI, CI and D_2_ were 0.76, 9.2%, 62.63 Gy for 1ARC; 0.73, 8.9%, and 62.55 Gy for 2TARC; 0.66, 11.2%, and 63.32 Gy for 4IMRT plans, respectively. For the volume PTVbreast, HI of 1ARC and 2TARC were statistically higher than that of 4IMRT plans. CI was best in 1ARC, then 2TARC and worst in 4IMRT. The differences in D_2_ and D_50_ were statistically insignificant for PTVbreast amongst the different techniques. The mean of Chair Index (CHI) in 1ARC (2.47) and 2TARC (2.62) were noticeably greater than that in 4IMRT (1.71).

**Table 1:** Dose coverage for the targets and the planning parameters (Plan A: 1ARC, Plan B: 2TARC, PlanC: 4IMRT, paired T test).

		Plan A	Plan B	Plan C	P value		
					A Vs B	B Vs C	A Vs C
PTVboost							
D_2_(Gy)	mean ± SD	65.08 ± 1.1	65.04 ± 0.37	66.60 ± 1.54	0.911	0.021	0.011
	max/min	67.15/ 62.84	65.71/63.77	69.43/63.99			
D_50_(Gy)	mean ± SD	62.63 ± 0.64	62.55 ± 0.10	63.32 ± 1.16	0.73	0.11	0.07
	max/min	63.89/61.79	62.69/62.35	65.15/62.31			
D_95_(Gy)	mean ± SD	60.11 ± 0.13	60.21 ± 0.21	60.27 ± 0.32	0.243	0.655	0.126
	max/min	60.29/60.01	60.40/60.01	60.64/60.01			
V107%	mean ± SD	8.55 ± 3.15	9.38 ± 2.56	16.7 ± 3.39	0.518	0.001	0.001
	max/min	13.01/3.52	12.3/3.4	19.8/11.5			
HI(%)	mean ± SD	9.2 ± 1.8	8.9 ± 0.7	11.2 ± 2.2	0.575	0.017	0.017
	max/min	12.6/7.2	10.1/7.9	15.5/9.7			
CI	mean ± SD	0.76 ± 0.1	0.73 ± 0.1	0.66 ± 0.16	0.059	0.022	0.008
	max/min	0.90/0.55	0.81/0.51	0.77/0.29			
PTVbreast							
D_2_(Gy)	mean ± SD	63.97 ± 0.80	63.91 ± 0.27	64.70 ± 1.45	0.24	0.76	0.46
	max/min	65.13/62.84	64.32/63.53	67.75/62.60			
D_50_(Gy)	mean ± SD	54.12 ± 0.72	53.87 ± 0.30	53.75 ± 1.06	0.85	0.18	0.29
	max/min	55.63/53.44	54.26/53.38	54.85/53.08			
D_95_(Gy)	mean ± SD	50.22 ± 0.19	50.10 ± 0.12	50.03 ± 0.08	0.057	0.214	0.051
	max/min	50.58/50.01	50.33/50.01	50.21/50.0			
V107%	mean ± SD	57.64 ± 6.90	55.90 ± 4.56	58.84 ± 4.0	0.330	0.233	0.732
	max/min	65.7/49	62/48.2	64.9/53.1			
HI(%)	mean ± SD	27.6 ± 1.9	27.8 ± 0.3	30.5 ± 2.7	0.82	0.02	0.01
	max/min	30.4/24.4	28.3/27.3	36.0/28.1			
CI	mean ± SD	0.75 ± 0.03	0.73 ± 0.04	0.72 ± 0.05	0.02	0.04	0.01
	max/min	0.80/0.71	0.78/0.64	0.79/0.62			
CHI	mean ± SD	2.47 ± 1.17	2.62 ± 0.91	1.71 ± 0.83	0.43	0.01	0.04
	max/min	3.84/0.87	3.95/1.76	3.2/0.72			
PTVbreast2*							
D_2_(Gy)	mean ± SD	60.66 ± 0.89	60.61 ± 0.48	61.1 ± 0.87	0.69	0.01	0.01
	max/min	62.53/59.84	61.34/59.96	62.97/60.16			
D_50_(Gy)	mean ± SD	53.47 ± 0.46	53.35 ± 0.26	53.74 ± 0.47	0.17	0.01	0.11
	max/min	54.19/53.02	53.62/52.86	54.56/53.21			
D_95_(Gy)	mean ± SD	50.22 ± 0.19	50.10 ± 0.12	50.03 ± 0.08	0.057	0.214	0.051
	max/min	50.58/50.01	50.33/50.01	50.21/50.0			
HI(%)	mean ± SD	23.67 ± 2.66	23.69 ± 1.17	25.41 ± 2.08	0.96	0.01	0.01
	max/min	28.53/20.51	25.66/22.24	29.6/23.2			
CI	mean ± SD	0.63 ± 0.03	0.61 ± 0.02	0.60 ± 0.02	0.01	0.01	0.01
	max/min	0.67/0.60	0.65/0.58	0.64/0.57			
CHI	mean ± SD	2.48 ± 1.17	2.63 ± 0.91	1.72 ± 0.83	0.44	0.03	0.04
	max/min	3.85/0.87	3.96/1.77	3.2/0.73			
Planning parameters							
Mus	mean ± SD	848.9 ± 86.6	817.5 ± 130.2	679.8 ± 76.6	0.460	0.009	0.000
	max/min	1000.9/741.7	1112.1/698.6	805.3/589.1			
Segments	mean ± SD	121.0 ± 7.2	106.9 ± 12.0	58.5 ± 1.51	0.066	0.006	0.003
	max/min	129/108	134/94	60/56			
Treatment time(s)	mean ± SD	123.7 ± 9.6	135.8 ± 16.7	269.4 ± 33.3	0.047	0.000	0.000
	max/min	137.3/107.1	162.8/120.2	294.5/193.0			

*PTVbreast2 is the combination volume of PTVbreast minus PTVboost.

The combination volume of PTVbreast minus PTVboost was denoted as PTVbreast2. For the volume PTVbreast2, HI of 1ARC and 2TARC were statistically better than that of 4IMRT plans. CI was highest in 1ARC, followed by 2TARC and the last 4IMRT. D_2_ were statistically higher in 4IMRT than that in 1ARC and 2TARC. The mean of Chair Index (CHI) in 4IMRT (1.72) was noticeably lower than that in1ARC (2.48) and 2TARC (2.63).

The average MUs and number of segments in 4IMRT (679.8 and 60, respectively) were remarkably less than that for 1ARC (848.89 and 121) and 2TARC (817.5 and 106.88). However, the treatment time with 4IMRT was twice as long as or more compared with 1ARC and 2TARC.

### 2TARC showed statistically lower radiation doses on Heart and Lung_Left when comparing with 4IMRT

**Figure 3:**
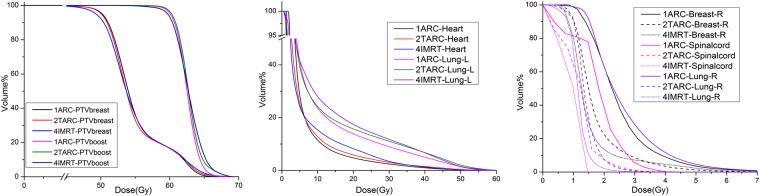
DVH curves for different treatment techniques (1ARC, 2TARC, 4IMRT) for PTVbreast, PTVboost, Heart, Lung_left, Lung_right, Breast_Right and Spinalcord.

It was shown in Table 2 that the V5 of Lung_Left and Heart are relative similar amongst the techniques, but the mean of V10, V20 and Dmean to Lung_Left and Heart in 2TARC (23.65%, 15.28%, 9.52 Gy and 13.46%, 7.23%, 5.38 Gy) were less than those in 4IMRT (28.10%, 17.54%, 10.20 Gy and 15.42%, 8.30%, 5.87 Gy, p < 0.05). Especially the mean dose to Heart in 2TARC was nearly 0.5 Gy lower than that in 4IMRT. The mean dose to Heart in 1ARC was close to that in 4IMRT. This can be also seen in the Fig. 3.

**Table 2:** Comparison of the doses to OARs (Plan A: 1ARC/Plan B: 2TARC/PlanC: 4IMRT, paried T test).

			Plan A	PlanB	Plan C	P value		
						A Vs B	B Vs C	A Vs C
Lung_Right	Dmean(Gy)	mean ± SD	2.58 ± 0.54	1.39 ± 0.18	1.15 ± 0.14	0.000	0.000	0.000
		max/min	3.33/1.77	1.74/1.21	1.34/0.95			
Spinal cord	Dmax(Gy)	mean ± SD	3.60 ± 0.86	2.16 ± 1.13	1.25 ± 0.17	0.000	0.000	0.000
		max/min	4.80/2.15	5.07/1.24	1.57/1.01			
Breast_Right	Dmean(Gy)	mean ± SD	2.60 ± 0.69	1.74 ± 0.38	1.51 ± 0.54	0.000	0.028	0.000
		max/min	4.08/1.82	2.38/1.42	2.50/1.06			
	Dmax(Gy)	mean ± SD	5.73 ± 1.66	4.95 ± 1.32	5.38 ± 2.06	0.023	0.289	0.246
		max/min	9.51/4.61	8.39/3.31	9.67/3.15			
Lung_Left	V5(%)	mean ± SD	41.22 ± 8.86	38.18 ± 4.45	41.73 ± 6.12	0.206	0.092	0.852
		max/min	56.5/33.6	46.4/33.5	51.7/34.6			
	V10(%)	mean ± SD	23.53 ± 4.52	23.65 ± 3.68	28.10 ± 5.22	0.895	0.008	0.004
		max/min	31/18.1	30.5/19.3	36.2/22.9			
	V20(%)	mean ± SD	13.83 ± 2.93	15.28 ± 2.92	17.54 ± 4.16	0.047	0.035	0.008
		max/min	18.5/9.6	20.5/11.5	26.2/12.1			
	Dmean(Gy)	mean ± SD	9.23 ± 1.51	9.52 ± 1.63	10.20 ± 1.90	0.348	0.036	0.009
		max/min	11.67/7.21	12.92/7.47	13.81/7.68			
Heart	V5(%)	mean ± SD	28.97 ± 9.62	23.84 ± 8.33	24.35 ± 7.29	0.070	0.936	0.225
		max/min	49.5/17.4	35.5/10.6	35.1/12.3			
	V10(%)	mean ± SD	11.43 ± 4.49	10.72 ± 2.90	15.42 ± 5.34	0.702	0.012	0.055
		max/min	19.1/7	15.2/6.58	21.9/7.3			
	V20(%)	mean ± SD	4.71 ± 1.72	5.41 ± 2.02	8.30 ± 3.89	0.525	0.008	0.003
		max/min	8.4/2.9	8.1/2.7	12.9/4.3			
	Dmean(Gy)	mean ± SD	5.83 ± 0.86	5.38 ± 1.01	5.87 ± 1.27	0.047	0.035	0.917
		max/min	7.33/5.05	6.81/4.18	8.32/4.20			
Body	V5(%)	mean ± SD	22.80 ± 4.05	17.04 ± 3.12	17.21 ± 2.67	0.000	0.784	0.000
		max/min	29.19/17.72	21.87/13.43	20.35/13.93

The mean doses (Gy) to Lung_Right, Spinal cord and Breast_Right in 4IMRT (1.15, 1.25,1.51) was lower than those in 1ARC (2.58, 3.60, 2.60) and 2TARC (1.39, 2.16,1.74) (Table 2). But the differences were quite small in these data between 4IMRT and 2TARC techniques. The max doses to Breast_Right were relative similar amongst the techniques (1ARC 5.73, 2TARC 4.95, 4IMRT 5.38 Gy).

In addition, the low dose volume V5 for Body was statistically higher in 1ARC (22.80%) than 2TARC (17.04%) and 4IMRT (17.21%).

### The doses to OARs increased and the plan quality deteriorated with inner quadrant tumors

Table 3 presents the comparison of OAR doses between with inner quadrant tumors and with non-inner quadrant tumors in 2TARC technique. The mean dose to the Heart, as well as Breast_Right, Lung_Right, Spinalcord, Lung_Left, was statistically higher with inner quadrant tumor than those with non-inner quadrant tumor. At the same time, HI, CI of PTVboost and CHI of PTVbreast were statistically worse with inner quadrant tumors than those with non-inner quadrant tumor (Table 3).

**Table 3:** Comparison of the doses to OARs and HI, CI for targets in 2TARC: the tumors in the inner quadrantvs in other quadrant of left breast (Paired test).

			Inner*	Non-inner*	All*	Inn Vs NI*	P values	Inn Vs All*
							NI Vs All*	
Lung_Right	Dmean(Gy)	mean ± SD	1.63 ± 0.10	1.31 ± 0.09	1.39 ± 0.18	0.000	0.171	0.014
		max/min	1.74/1.52	1.40/1.21	1.74/1.21			
Spinal cord	Dmax(Gy)	mean ± SD	4.41 ± 0.58	1.41 ± 0.09	2.16 ± 1.13	0.001^+^	0.281^+^	0.004
		max/min	5.07/3.75	1.46/1.24	5.07/1.24			
Breast_Right	Dmean(Gy)	mean ± SD	2.33 ± 0.04	1.54 ± 0.11	1.74 ± 0.38	0.001^+^	0.281^+^	0.020^+^
		max/min	2.38/2.27	1.72/1.42	2.38/1.42			
	Dmax(Gy)	mean ± SD	7.28 ± 1.01	4.18 ± 0.57	4.95 ± 1.32	0.001^+^	0.330^+^	0.011
		max/min	8.39/6.17	4.96/3.31	8.39/3.31			
Lung_Left	V5(%)	mean ± SD	44.68 ± 1.46	36.02 ± 1.99	38.18 ± 4.45	0.000	0.231	0.006
		max/min	46.4/42.96	38.5/33.5	46.4/33.5			
	V10(%)	mean ± SD	28.12 ± 2.01	22.17 ± 2.46	23.65 ± 3.68	0.001	0.646^+^	0.028
		max/min	30.5/25.73	26.4/19.3	30.5/19.3			
	V20(%)	mean ± SD	19.98 ± 2.41	14.38 ± 2.34	15.28 ± 2.92	0.023	0.554	0.108
		max/min	20.5/15.45	18.5/11.5	20.5/11.5			
	Dmean(Gy)	mean ± SD	11.42 ± 1.28	8.89 ± 0.95	9.52 ± 1.63	0.001	0.646^+^	0.044
		max/min	12.92/9.92	10.34/7.47	12.92/7.47			
Heart	V5(%)	mean ± SD	30.65 ± 4.11	21.57 ± 7.97	23.84 ± 8.33	0.051	0.509	0.13
		max/min	35.5/25.8	33.9/10.6	35.5/10.6			
	V10(%)	mean ± SD	12.73 ± 0.34	10.05 ± 3.09	10.72 ± 2.90	0.078^+^	0.532	0.152^+^
		max/min	13.1/12.35	15.2/6.58	15.2/6.58			
	V20(%)	mean ± SD	5.97 ± 1.75	5.22 ± 2.0	5.41 ± 2.02	0.41	0.803	0.493
		max/min	7.95/3.99	8.1/2.7	8.1/2.7			
	Dmean(Gy)	mean ± SD	6.42 ± 0.36	5.03 ± 0.89	5.38 ± 1.01	0.007	0.384	0.027^+^
		max/min	6.81/6.03	6.48/4.18	6.81/4.18			
PTVboost	HI(%)	mean ± SD	9.78 ± 0.32	8.61 ± 0.37	8.9 ± 0.7	0.000	0.275	0.005
		max/min	10.12/9.44	8.90/7.92	10.12/7.9			
	CI	mean ± SD	0.62 ± 0.09	0.77 ± 0.03	0.73 ± 0.1	0.001^+^	0.232	0.041
		max/min	0.72/0.51	0.81/0.72	0.81/0.51			
PTVbreast	HI(%)	mean ± SD	27.97 ± 0.01	27.72 ± 0.33	27.8 ± 0.3	0.078^+^	0.855	0.124^+^
		max/min	27.97/27.98	28.27/27.35	28.3/27.3			
	CI	mean ± SD	0.69 ± 0.05	0.74 ± 0.03	0.73 ± 0.04	0.075^+^	0,388	0.153
		max/min	0.74/0.64	0.78/0.70	0.78/0.64			
	CHI	mean ± SD	1.87 ± 0.11	3.23 ± 1.02	2.62 ± 0.91	0.005^+^	0.638	0.012^+^
		max/min	1.98/1.76	3.95/2.72	3.95/1.76			
PTVbreast2^#^	HI(%)	mean ± SD	25.3 ± 0.31	23.05 ± 0.59	23.69 ± 1.17	0.016^+^	0.759	0.021^+^
		max/min	25.66/24.94	23.78/22.24	25.66/22.24			
	CI	mean ± SD	0.59 ± 0.01	0.62 ± 0.02	0.61 ± 0.02	0.059^+^	0.403	0.121
		max/min	0.60/0.58	0.65/0.58	0.65/0.58			
	CHI	mean ± SD	1.83 ± 0.05	2.95 ± 0.94	2.63 ± 0.91	0.009^+^	0.526	0.015^+^
		max/min	1.88/1.77	3.96/1.77	3.96/1.77			

*Inn, Inner: the inner quadrant tumors; Non-inner, NI: the non-inner quadrant tumors; All: all the tumors.

+The variances of two group tested are not equal at the significant level 0.10, so the Wilcoxon rank sum test is used.

#PTVbreast2 is the combination volume of PTVbreast minus PTVboost.

## Discussion

The choice of radiation technique is very important for the patients with early stage breast cancer, for their long life expectancy after the treatment. The radiation induced diverse effects would happen ten years or more after the treatment which cannot be totally remedied. In this study, the aim was to find the optimal technique for WBI SIB amongst the three techniques: 1ARC, 2TARC, 4IMRT.

We can find from Table 1 that the planning aim for the Target coverage was achieved for all treatment plans. HI and CI of PTVboost in VMAT plans were better than 4IMRT plans (p < 0.05). And D2 of PTVboost in 1ARC and 2TARC were statistically lower than that in 4IMRT. This might reduce the rate of breast fibrosis and improve the cosmetic outcomes. These results were same in the studies of Wu *et al*.^7^ and Aly *et al*.^8^ Wu *et al*.^7^ reported that CI of PTVboost in VMAT (0.91) was obviously higher than that in IMRT (0.84) but the HIs of PTVboost were same in two plans. In the study of Aly *et al*.^8^, the CI of PTVboost in one partial arc VMAT was equal to that of other plans but the HI of PTVboost in one partial arc VMAT was better than that of other plans.

In this study Chair Index (CHI) was introduced to assess the dose homogeneity in SIB. Generally, HI is used to describe the dose homogeneity in a target volume. However, in the case of SIB with multiple dose levels the use of HI becomes problematic. The high dose tail in the lower dose target elevates the HI value, and insensitive to the qualitative difference in the dose distribution. For example, the HI of PTVbreast were 27.6 ± 1.9 for 1ARC, 27.8 ± 0.3 for 2TARC, and 30.5 ± 2.7 for 4IMRT respectively, and the differences of HI between VMAT and 4IMRT were statistically significant (p < 0.05). CHI was designed for the chair-shaped DVH curve that for SIB. A higher CHI means better dose distribution in the lower dose target (excluding the boost volume). It was shown in Table 1 that mean CHI in 1ARC (2.47) and 2TARC (2.62) were statistically higher than in 4IMRT (1.71), which indicates the dose distribution in PTVbreast was improved in VMAT plans.

In our study the boost volume was total included by the target volume. However, the boost volume was cropped form the target volume in the study of Aly *et al*.^8^ So the target volume was the combination volume of PTVbreast minus PTVboost (PTVbreast2) in this study. For the volume PTVbreast2, HI of 1ARC and 2TARC were statistically better than that of 4IMRT plans. CI was best in 1ARC, then 2TARC and worst in 4IMRT. D_2_ were statistically higher in 4IMRT than that in 1ARC and 2TARC. The average CHI in 4IMRT (1.72) was still statistically lower than that in1ARC (2.48) and 2TARC (2.63). All these data told us that the dose distribution in PTVbreast2 was better in 1ARC and 2TARC than in 4IMRT.

In this study CHI had shown its effectiveness in assessing the dose heterogeneity in the target containing a boost volume. We must select the dose values L, M and H cautiously, especially L. V_L_ were located in the first fall-off of the target cumulative DVH curve. CHI might fail with the inappropriate L, M and H. Future work will be to test CHI for other treatment sites with SIB.

The life expectancy of patients after BCS was usually longer than 10 years. Therefore the doses to the OARs should be kept as low as possible. Darby *et al*.^18^ estimated that the risk of major coronary events increases linearly 7.4% per Gy in the mean radiation dose to the heart. Nitsche *et al*.^19^ reported that cardiovascular diseases were the leading cause of death in women of USA. In our study we found out that: V5 of Heart in 1ARC was notably higher than that in other two plans; V10 and V20 of Heart in 1ARC were close to those in 2TARC, but lower than those in 4IMRT; the Dmean to Heart in 2TARC (5.37 Gy) was statistically lower than that in 1ARC (5.83 Gy) and 4IMRT (5.86 Gy). Overall, the 2TARC was the best plan to heart amongst three studies techniques. This result was contradicting with the finding of Wu *et al*.^7^ which reported that V5, V10 and Dmean of Heart in VMAT were significantly higher than those in IMRT. The reported values for the mean Heart V20 were relatively higher for both VMAT and IMRT when compared to our study. The different plan designs and treatment machine might be the reasons for it. The 5mm leaf width (at isocenter) of Axesse linac used in our study was helpful to improve the plan quality. Aly *et al*.^8^ reported the maximum of Dmean to Heart 4.2 Gy. The minimum Dmeans to heart were 4.97 Gy in Wu *et al*.’s^7^ and 5.37 Gy in our study. Tumor in the inner quadrant would increase the irradiation to the Heart, both Lung and Breast_Right (Table 3). We have shown that the location of the boost volume may have a significant impact on the OAR dose, which may at least partly explain the differences in the OAR doses between the different reports.

In comparison the dose to Lung_Left, 4IMRT techniques did not show any advantages over two other techniques. The V10, V20 and Dmean of Lung_Left in 4IMRT were statistically higher than those in 1ARC and 2TARC. This result was also contradicting the studies of Wu *et al*. and Aly *et al*. In their studies, the doses to Lung_Left were higher in VMAT than in IMRT.

As for dose to other OARs, the results were similar in all studies. The doses to Lung_Right, Breast_Right and Spinal cord in 4IMRT were lowest, the next in 2TARC and the highest in 1ARC. However, the dose differences between in 2TARC and in 4IMRT were very small in our study. For example, the Dmean to Breast_Right and Lung_Right in 4IMRT were 1.51 and 1.15 Gy, only 0.23 and 0.24 Gy lower than those in 2TARC respectively. It was hard to evaluate the clinical meaning of those small values.

VMAT has the dosimetric advantage over the IMRTas there is no limit in the direction of beam incidence. However, this advantage comes with the cost of increased low dose volume (<5 Gy). In our study, the mean Body V5 in 1ARC was statistically higher than that in 2TARC and 4IMRT, and the values in latter two were very close. IMRT and VMAT had their own characteristics and Radiation oncologists must balance their merits and demerits before using them. In our study, the doses to OARs were strikingly lowered by changing a large arc (1ARC) into two small tangential arcs (2TARC). Meanwhile, the dosimetric parameters for targets were nearly the same between 1ARC and 2TARC. 2TARC was shown to be the best amongst three techniques. 2TARC had better HI, CI and CHI for targets than 4IMRT plans while nearly the same doses to the OARs–the dose to Heart in 2TARC was lower than those in 4IMRT and 1ARC.

In conclusion, 2TARC was shown to be the optimal treatment technique amongst the studied techniques for patients with left-sided breast cancer after BCS, if they chose the photon therapy. The doses to OARs were shown to increase significantly for the patients with inner quadrant tumor.
